# Description of a New Predictive Modeling Approach That Correlates the Risk and Associated Cost of Well-Defined Diabetes-Related Complications With Changes in Glycated Hemoglobin (HbA1c)

**DOI:** 10.1177/1932296816662048

**Published:** 2017-03-01

**Authors:** Kurt Fortwaengler, Christopher G. Parkin, Kurt Neeser, Monika Neumann, Oliver Mast

**Affiliations:** 1 Roche Diabetes Care GmbH, Mannheim, Germany; 2 CGParkin Communications, Inc, Boulder City, USA; 3 LASER Analytica, Lörrach, Germany; 4 Roche Diagnostics GmbH, Mannheim, Germany

**Keywords:** diabetes, HbA1c, model, approach, cost, complications

## Abstract

The modeling approach described here is designed to support the development of spreadsheet-based simple predictive models. It is based on 3 pillars: association of the complications with HbA1c changes, incidence of the complications, and average cost per event of the complication. For each pillar, the goal of the analysis was (1) to find results for a large diversity of populations with a focus on countries/regions, diabetes type, age, diabetes duration, baseline HbA1c value, and gender; (2) to assess the range of incidences and associations previously reported. Unlike simple predictive models, which mostly are based on only 1 source of information for each of the pillars, we conducted a comprehensive, systematic literature review. Each source found was thoroughly reviewed and only sources meeting quality expectations were considered. The approach allows avoidance of unintended use of extreme data. The user can utilize (1) one of the found sources, (2) the found range as validation for the found figures, or (3) the average of all found publications for an expedited estimate. The modeling approach is intended for use in average insulin-treated diabetes populations in which the baseline HbA1c values are within an average range (6.5% to 11.5%); it is not intended for use in individuals or unique diabetes populations (eg, gestational diabetes). Because the modeling approach only considers diabetes-related complications that are positively associated with HbA1c decreases, the costs of negatively associated complications (eg, severe hypoglycemic events) must be calculated separately.

Several sophisticated models have been developed to assist researchers and policy makers in predicting complications survival and medical care costs in both individuals with diabetes and representative diabetic populations.^1-9^ However, because these models are complex and require multiple input variables in their calculations (eg, IMS Core Diabetes Model [IMS CDM],^6^ IMIB model,^8^ Archimedes^9^), their utilization outside of research settings is not always practical. Moreover, the mathematical foundations and data sources utilized are often not sufficiently transparent.

Simple health-economic models used for publications often consist of only 1 source per complication.^10,11^ Such models are simple to produce and use but bear the risk of relevant over- or underestimations.

The approach for predictive modeling was designed to meet the highest possible ease of use and transparency but also satisfying reliability. This article presents a description of an approach that helps to associate the risk and associated cost of well-defined diabetes-related complications with changes in glycated hemoglobin (HbA1c). The goal was to develop a flexible, transparent approach to modeling that allows clinicians, payers, and public policy makers to quickly obtain reliable estimates of costs and complication incidences in their specific populations without complex handling of several input variables.

## Methods

### Overview of the Modeling Approach

The new modeling approach is a Microsoft Excel spreadsheet-based tool, designed to support the development of simple predictive models that estimate the health and economic impact of changes in HbA1c values. It is intended for use in average insulin-treated diabetes populations with baseline HbA1c values within an average range (6.5% to 11.5%); it is not intended for use in individuals or unique diabetes populations (eg, gestational diabetes) or over an extended time span in populations with relatively short remaining life spans. Because the modeling approach only considers diabetes-related complications that are positively associated with HbA1c decreases, the costs of negatively associated complications (eg, severe hypoglycemic events [SHE]) must be calculated separately.

The modeling approach is based on 3 pillars: association of the complications with HbA1c changes, incidence of the complications, and average cost per event of the complication. To ensure the quality of the modeling approach, a comprehensive, systematic review of the literature was conducted to assess the range of incidences and associations previously reported. For each pillar, the goal of the analysis was to find results for a large diversity of populations with a focus on countries/regions, diabetes type, age, diabetes duration, baseline HbA1c value, and gender.

The range of incidences and associations previously reported were assessed and documented. As result, the user is warned when extreme figures (eg, prevalence instead of incidences) are inserted by comparing them with the realistic range of previously reported data.

### Literature Review

The systematic review was conducted between June 10 and 15, 2013, on the DIMDI platform by using following databases: Medline, Embase/Embase Alert, Cochrane Central Register of Controlled Trials, Cochrane Database of Systematic Reviews, Database of Abstracts of Reviews of Effects, Health Technology Assessment Database, NHS EED. The search was performed with a language restriction (including English and German). Three searches were performed. Search 1 identified relevant references that allowed for quantification of the relationship between HbA1c level and the risk of short- and long-term complications and hospital admissions published between 1990 and June 2013. Of primary interest were microvascular complications (retinopathy, neuropathy, nephropathy), macrovascular complications (acute myocardial infarction [AMI], angina, stroke), and miscellaneous complications (amputation, depression, others). Search 2 focused on the incidence rates of the diabetes-related complications that were identified in search 1 with no restrictions with regard to countries published between 2003 and June 2013. In this search, incidence of complications and the impact of patient characteristics were considered. Search 3 retrieved information about treatment costs of complications that were identified in search 1 with no restrictions with regard to countries published between 2003 and June 2013. Costs for treatment of short- and long-term complications, hospitalizations and outpatient visits were considered.

Study selection was performed by 1 reviewer at the title, abstract and full-text levels to identify relevant articles against predefined inclusion/exclusion criteria, which included but were not limited to the PICO (Problem/population, Intervention, Comparison and Outcome) elements.^12^ Specifically, eligible reports included type 1 diabetes (T1DM) or type 2 diabetes (T2DM) populations. Insulin treatment was mandatory for search 1 and search 2 but not for search 3 (costs). A single failed eligibility criterion was sufficient for a study to be excluded from the review. Uncertainties and ambiguities regarding abstract and full-texts were resolved through consensus adjudication.

**Figure 1.**
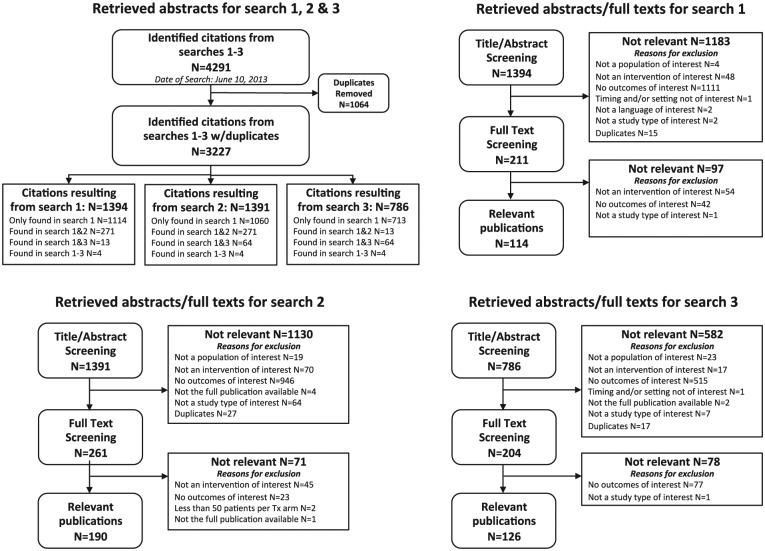
Flow diagram of reviewed studies. (A) The literature search in bibliographic databases retrieved a total of 4291 citations for all 3 research questions. After electronic removal of 1064 duplicates, 3227 abstracts remained for screening against inclusion/exclusion criteria. (B) The search for sources describing the relationship of HbA1c level and diabetes-related complications (search 1) provided a total of 1394 citations for the screening against inclusion/exclusion criteria. Out of these 1183 abstracts were excluded and 211 citations were found eligible for the screening on full-text level. Of these full-text publications, 97 articles were excluded and 114 were found eligible. The majority of the eligible publications provided relationship results concerning the most common complications. Relationship results concerning nephropathy, retinopathy, cardiovascular diseases, hypoglycemia, and neuropathy were found in 47, 22, 15, 14, and 10 publications. The other complications were found in relatively few publications. (C) The search for sources with regard to incidence rates of diabetic complications (search 2) provided a total of 1391 citations for the screening against inclusion/exclusion criteria. Out of these, 1130 abstracts were excluded and 261 citations were reviewed on the full-text level. In total, 190 full-text articles were considered as useful for this research questions and 71 articles were excluded. The majority of the eligible publications provided results with regard to incidences of the most common complications: hypoglycemia (n = 105), nephropathy (n = 64), cardiovascular diseases (n = 61), and retinopathy (n = 49). (D) The search for sources focusing on diabetes-related complication costs (search 3) resulted in 786 citations for the screening against inclusion/exclusion criteria. Of these, 582 abstracts were excluded and 204 citations were reviewed on full-text level. Seventy-eight articles were excluded and 126 were found eligible. The majority of eligible publications provided cost results concerning all diabetes-related complications (n = 43) or several complications bundled together (n = 24). The cost results concerning nephropathy revealed 18 publications while 16 publications have cost results attributed to hypoglycemia. Hospitalization costs for diabetes management were found in 18 publications. The other complications were found in relatively few publications.

Figure 1 presents a flow diagram of the studies reviewed for inclusion in the modeling approach. Table 1 presents the number of publications identified for the various diabetes-related complications by search.

**Table 1.** Number of Publications for Various Diabetes-Related Complications.

Complication	Search 1 (relationship)	Search 2 (incidences)	Search 3 (costs)
Cardiovascular	15	61	6
Nephropathy	47	64	18
Retinopathy	22	49	2
Hypoglycemia	14	105	16
Ketoacidosis	1	12	1
Cataract	1	3	
Amputation/ulcer	6	11	2
Mortality	6	14	
Hospitalization	5	8	18
Neuropathy	10	11	
Pregnancy/birth	5	15	
All diabetes-related complications	1		43
Several diabetes-related complications	2		24
Diabetes-related endpoint		1	
Erectile dysfunction	1		
Hyperglycemia		1	
Limited joint mobility	1		
Seizure/coma		2	

### Selection of Diabetes-Related Complications for Inclusion

Only diabetes-related complications with useable data for all 3 pillars could be included in the modeling approach. In addition, data independent from other complications were needed. Therefore, hospitalizations were not included. Most considered complications include some time in hospital. Thus, the cost of hospitalizations is partly covered by the cost of the other complications. This is also true for mortality. Based on these criteria, the following diabetes-related complications are included in the modeling approach:

- Cardiovascular complications: angina, coronary heart failure (CHF)
- Myocardial infarction (MI)
- Brain complications: stroke, diabetic ketoacidosis (DKA)
- Eye diseases: proliferative retinopathy (PR), severe vision loss (blindness)
- Peripheral complications: amputation, neuropathy
- End-stage renal disease (ESRD)

### Identification and Documentation of Previously Reported Effect Sizes and Incidences

#### Effect sizes

Only publications reporting study size and duration were considered. That allowed use of a weighted average based on person years. Because many of the studies that provided associations were of long duration (average 8.9 years), baseline or average HbA1c values (which had changed during the study period) were not reported in all studies. Fifty-one of the 118 analyzed publications with associations were useable for calculations. Of those, approximately 50% delivered average HbA1c values (range: 6.5-11.8%, average: 8.29%).

To combine data from the multiple sources, the relative change per 1% HbA1c reduction (the most commonly used deviation in the studies analyzed) were calculated. Then we calculated an “average deviation”. It was not possible to perform a full meta-analysis because of the conversions, which eliminated information (eg, *P* values, confidence intervals). Thus, the goal was to identify the deviation closest to the average and median of all deviations found.

For some of the complications (eg, DKA, neuropathy, PR), there were not enough studies to represent the full range of the T1DM and T2DM populations. For the search of effect sizes in long-term complications, life expectancy needed to be as high as possible and the prevalence as small as possible (best case: zero) in the baseline population. This usually only can be found in children. In such cases, the associations found for the T1DM patients were used as proxy of the effects on insulin-treated T2DM patients when other evidence suggested similar effect sizes for both populations. This was considered to be acceptable because the model is intended for use with insulin-treated diabetes only.

#### Incidences

Because some of the complications happen only once (eg, ESRD, blindness), the “incidence” of these complications was considered to be the number of new cases per year in the population without that complication. With complications that can occur more than once (eg, MI, DKA), the total number of occurrences was calculated. Thus, the “incidence” is the number of cases per patient year in the overall population.

In the evidence analyzed that reported incidences, the range of baseline or average HbA1c values was 6.4% to 13.5% (average 8.61%). Identified complication incidences were normalized to a “default” HbA1c value of 8.56% (the average within the selected publications that reported incidence data), using the identified effect sizes. Although this had some effect on the average incidences of each single complication, the effect on the overall results is very limited: the overall number of cases was increased by <1%, the number of potentially prevented events was increased by ≈0.5%, the total cost of all complications was decreased by ≈2.4%, and the potential cost savings were decreased by ≈2.1%.

Usage of the normalized incidences for each complication ensured that the strongest influencing factor was minimized. However, similar to the calculations of associations (discussed earlier) this prevented performing a meta-analysis because of the normalization, which eliminated relevant information such as *P* values and confidence intervals. In addition, calculating a weighted average was not meaningful. For example, using a large study with over 600 000 patients that also included non-insulin-treated patients,^13^ would have been dominant.

#### Estimated Costs

In the cost analysis, the direct cost of complications was sought. For the complications with ongoing cost (eg renal disease with dialysis), the cost used in the model is the continuing cost over N years, where the N can be defined by the model user. For other complications, the cost of an “event” was used. This included the additional cost over the next N years, when available.

In most cases, the cost of a given complication was found to be relatively independent of the individuals studied. It is understood that hospitalizations of older patients or patients with more comorbidities or higher HbA1c values tend to be longer than that of younger or healthier patients, which would result in higher costs; however, the effects of those differences were small in comparison to other influencing factors.

The cost of a complication is always country-specific data. The user should use local cost data that can be inserted into the model. If not available, the approach contains 16 complete cost data sets from several countries, including Australia,^14^ Canada,^15,16^ China,^17^ Czech Republic,^18^ Germany,^19^ Saudi-Arabia,^20^ Sweden,^21,22^ Switzerland,^23^ Thailand,^24^ the United Kingdom,^25,26^ and the United States.^27-29^ The user can select 1 of those cost data sets. When another cost data set is used, the 16 existing cost data sets can serve as comparator (to verify the cost data).

More than 1 data set was found for 4 countries (Canada, Sweden, the United Kingdom, and the United States), which were used to perform comparisons of the results within each country (see online Appendix V).

#### Documentation

For each complication, the range of found incidences and effect sizes identified in the systematic literature review (separated by diabetes type) is documented in the model. They are also documented in the online Appendices (Appendices II, III).

#### Principle Behind Calculation/Utilization of Baseline Incidences Associated With HbA1c

In the systematic literature search, a variety of approaches to representing the association of HbA1c and incidence of complications was found. The 3 most common deviations were:

- Relative percentile change of incidence per *absolute* HbA1c change. With every 1% absolute reduction of the HbA1c value, the incidence decreases by a certain (constant) percentage. The absolute effect is larger for higher HbA1c values because here, the incidence is higher.
- Relative percentile change of incidence per *relative* HbA1c change. With every 10% relative reduction of the HbA1c value, the incidence decreases by a certain (constant) percentage. Again, the absolute effect is larger for higher HbA1c values because the incidence is higher. However, although the deviation shows a pattern similar to the one above, the effect is slightly smaller in the higher HbA1c ranges and slightly larger in the lower HbA1c ranges.
- Individual formula basing on the odds ratio (OR) and the Euler function. In these studies, several other factors (eg, blood pressure, blood lipids, smoking, etc) were considered in addition to the HbA1c value.

For the approach, the relative change of incidence per absolute HbA1c change was selected because it is the most common and least complex deviation to manipulate. For the publications with one of the other deviations, the “best fit” trend line of type “percentile change per 1% HbA1c reduction” was identified on an individual basis. However, this could be considered in the approach only if it led to a deviation very close to the original one. (see online Appendix I, IV)

**Figure 2.**
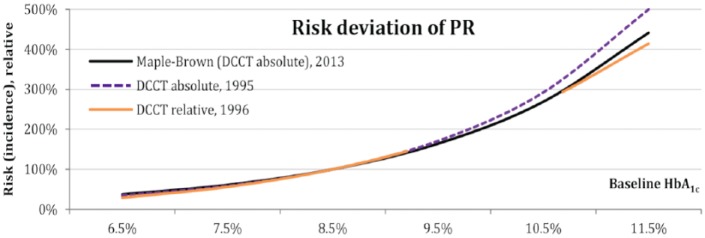
Three different deviations found for the same data (DCCT patients) by 3 analysts.^31-33^

Sometimes different publications from the same study show different deviations. The well-known DCCT study is a good example of this.^30^ Using the DCCT data, different investigators looked at retinopathy risk in the DCCT population.^31-33^ In these publications, 3 different trend lines were found. However, each trend line appeared to be a good proxy for the others. As shown in Figure 2, the trend line reported by Maple-Brown^31^ appears to be a good representative for the 3 publications. As such, it was used to illustrate the DCCT data in the modeling approach.

## Diabetes Type Specific Versus Mixed Population Model

Of particular in interest is the total number and cost of complications of all types covered by the approach for a given population. With this information, it is possible to directly calculate the total number and cost of prevented events, which is the main outcome of the approach.

The modeling approach contains ranges for both T1DM and T2DM populations. For a comparison, the averages of both data sets were used for calculation in the example model. The observed differences in number and cost of prevented events were surprisingly small. To address this, a mixed population data set was added, preferring results from meta-analyses or studies on mixed populations. The calculation with the averages of the mixed population data set, again, led to very similar results, with a trend to an underestimation of the effects.

## Sensitivity Analysis

To test the integrity of the approach, a sensitivity analysis was conducted, looking at T1DM values only, T2DM values only, combined T1DM and T2DM values, and the mixed population model values. The baseline calculation applies to a population with an average HbA1c value of 8.5% and an average HbA1c reduction of 0.5%. All variables without baseline HbA1c and diabetes type were varied by ±10% (each single incidence, association or cost). For the diabetes type, the results of the mixed population model were compared with the results of models based on T1DM or T2DM data. For the baseline HbA1c, the value was varied by ±0.5% absolute, which is ±10% of the supported range, 6.5% to 11.5%.

As presented in Table 2, the results of the HbA1c translator model for a mixed population tend to be underestimations. The only outcomes without underestimation are total cost of complications (12.6% lower for T1DM patients) and potential cost savings (3.3% lower for T2DM patients). However, because several complications with impact on the overall result (eg, other states of eye and kidney disease) were excluded, overestimation was minimized or avoided. For all improvements (prevented events, cost savings), there is an underestimation of approximately 5-10% compared with a T1DM or the average population. Because of the adaption of the baseline risks to the baseline HbA1c value, the model can be used in the full range of supported HbA1c values.

**Table 2.** Sensitivity Analysis Using Unadjusted and Adjusted (Normalized) Incidences Within a German Cost Setting.

Unadjusted incidences	T1DM average	T2DM average	Total average	Mixed population
Total incidence	0.126	0.168	0.149	0.124
Total prevented events per 1% HbA1c reduction	0.036	0.036	0.039	0.033
Total cost (German cost setting) (€)	2486	4161	2927	2785
Total cost savings per 1% HbA1c reduction (€)	569	511	572	539
Adjusted incidences				
Total incidence	0.125	0.175	0.154	0.124
Total prevented events per 1% HbA1c reduction	0.036	0.038	0.041	0.033
Total cost (German cost setting) (€)	2377	4023	2882	2721
Total cost savings per 1% HbA1c reduction (€)	547	511	566	528

In the analysis it was found that the number of prevented complications remains almost unchanged when the proportion of T2DM patients varies. The potential cost savings are influenced by the proportion of T2DM patients but are mostly underestimating. However, for a pure T2DM population, the results of the mixed population model are ≈3.3% higher than the results of the T2DM model.

**Figure 3.**
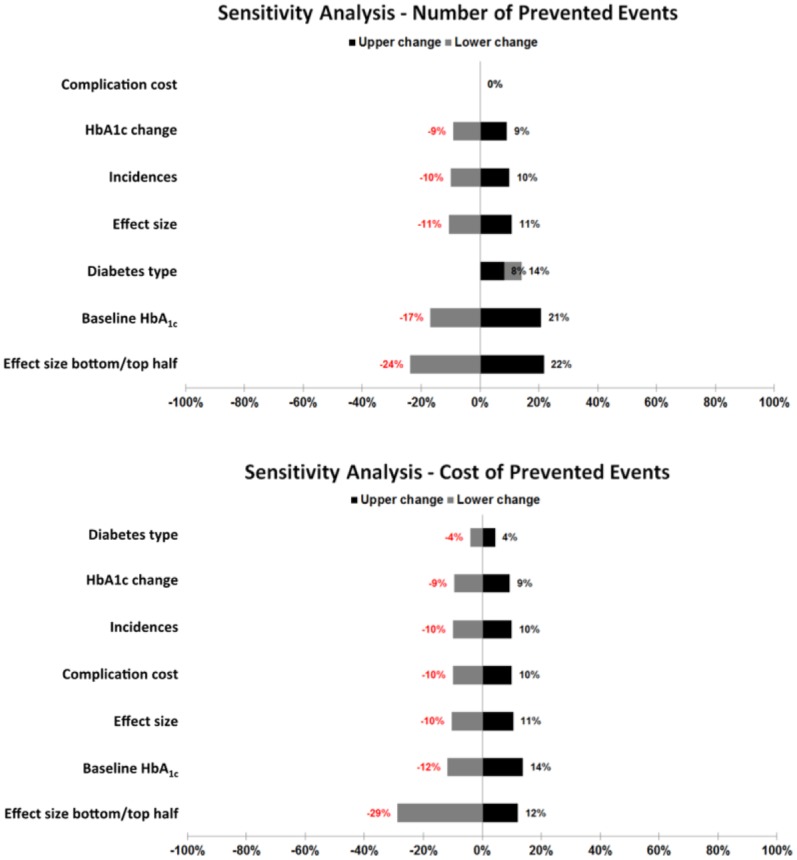
Sensitivity analyses for number and cost of prevented events.

As shown in Figure 3, one can see the expected results when comparing the impact of several influence factors on the overall results (number and cost of prevented events): (1) changes in the cost of events have no effect on the number of prevented events and a linear impact on the cost of prevented events, (2) changes in the incidences of events show the expected linear influence on both number and cost of prevented events, (3) changes in the HbA1c change and associations show the expected nonlinear influences, (4) the baseline HbA1c shows the expected high nonlinear influence, and (5) although the diabetes type shows some influence on the cost of prevented events, the effect is moderate in comparison to all other influence factors and always underestimates the number of prevented events.

This demonstrates that the modeling approach can be used with mixed population of both T1DM and insulin-treated T2DM, regardless of age, gender, and other demographic parameters. However, it is not appropriate for use in purely special populations (eg, pediatric, gestational) or single individuals.

The modeling approach contains 16 cost data sets. More than 1 source was found for some countries, including Canada,^15,16^ Sweden,^21,22^ the United Kingdom,^25,26^ and the United States.^27-29^ In comparisons to determine the influence of selecting an appropriate source, results showed that the selection of the appropriate source has some influence, but in most cases, it is very limited. For more details, see Appendix V.

## Utilization of the Modeling Approach

The modeling approach is designed for ease of use and flexibility, and it is intended for predictive modeling on insulin-treated diabetes patients. Based on this approach, several study-specific applications will be produced to demonstrate the clinical and cost impact of various interventions used in clinical trials. The “HbA1c Translator,” the model used to calculate the figures shown in this publication, is the first model built with approach. For more details, see Appendix VI.

## Discussion

This modeling approach is distinguished from other models by several features. Unlike the sophisticated models, which utilize multiple input variables in their calculations,^6,8,9^ this approach only requires input of the size and baseline HbA1c value of the population to be studied. Although the country-specific default settings streamline use of the modeling approach, users can easily adjust all data inputs to more precisely reflect individual circumstances. However, unlike typical simple predictive models, the modeling approach described here provides figures that allow the user to see how his/her selection fits to the identified range.

To illustrate the need for such a comparison, a “best case” (all incidences and associations using the maximum found in the literature) versus “worst case” (all incidences and associations using the minimum found in the literature) scenarios for a baseline population of 1000 German patients was calculated. The expected events varied between 21.44 and 413.60 (vs 122.00 for mixed population model) per year while the expected cost of events varied between €341 625 and €7 471 501 (vs €2 685 170). The expected effect of a 0.5% reduction in the average HbA1c value varied between 1.82 (8.5%) and 81.74 (19.8%) cases (vs 17.62 [14.4%]), while the expected cost of prevented events varied between €20 999 (6.1%) and €1 082 677 (14.5%) (vs €276 344 [10.3%]). Thus, a simple predictive model bears the risk of possible variations of around 2000-5000%, depending on the selection of evidence used.

A key limitation of the modeling approach is that the risk and cost predictions are not as accurate as the sophisticated models. However, sensitivity analyses verified that the modeling approach described here tends to underestimate the effects of HbA1c reductions, which ensures that the actual effects will likely be greater than the predicted effects.

It is important to reiterate that the approach is intended for use in average insulin-treated diabetes populations, with baseline HbA1c values within a defined average range. For calculations of individuals or special diabetes populations, individual models built for that purpose or one of the sophisticated models (eg, IMS Core Diabetes Model [IMS CDM],^6^ IMIB model,^8^ Archimedes^9^) are mandatory and require several additional input values.

Because life expectancy and utilities are not considered, it is not possible to calculate often-requested results such as quality-adjusted life years (QALYs). It is also not possible to calculate statistical values such as confidence intervals and *P* values. Because information about the therapies used to reach the HbA1c reduction is not queried, it is not possible to estimate the number of side effects of the HbA1c reduction (eg, SHE). Whenever the approach is used to build a model, the effect of the HbA1c change on the SHE must be examined.

## Conclusions

In summary, the approach allows users to model reliable estimates of risk and cost changes associated with HbA1c changes in insulin-treated diabetes populations. The approach draws on medical evidence based on studies of over 1.08 million patients or 9.6 million patient years. The information resulting from this modeling approach may assist clinicians, payers and public policy makers to more effectively and efficiently allocate health care resources based on ad hoc assessment. A key strength of the modeling approach is that it can be used for both T1DM and insulin-treated T2DM populations.

## Supplementary Material

Supplementary material
