# Supplementary material for: Description of a New Predictive Modeling Approach That Correlates the Risk and Associated Cost of Well-Defined Diabetes-Related Complications With Changes in Glycated Hemoglobin (HbA1c)
Source: J Diabetes Sci Technol. 2017 Mar 1;11(2):315–23. doi: 10.1177/1932296816662048 (PMC5478016; doi:10.1177/1932296816662048)
Supplement: Supplementary material [file FINAL_-_FortwaenglerAppendices.docx]

**APPENDICES**

**Appendix I: How to Determine the ‘Average Deviation’ (Example)**

To demonstrate how results from the studies were reconciled, the following example is presented, which includes results from five fictitious studies with different average baseline values and different baseline incidences. (**Figure 1A**) Three of the studies utilized *absolute* HbA_1c_ change and two utilized *relative* HbA_1c_ change.

**Figure 1A** Example of risk deviation in five fictitious studies.

The average HbA_1c_ value was approximately 8.5%. Because all five studies covered this HbA_1c_ value, it was possible to calculate the ‘normalized’ incidence for each of the studies then calculate the average incidence for our average HbA_1c_ value as indicated in **Figure 1B** (the red dot).

**Figure 1B**

It was also possible to replace the deviations other than ‘percentile change per 1% HbA_1c_ reduction’ with the proxies of this type. Using the same calculated ‘average incidence’ for each of the deviations transforms them such that they cross the one ‘average’. (**Figure 1C**) The red curve shows the deviation with the average percentile change (effect). A weighted average was used to limit the influence of the studies with extreme populations.

**Figure 1C**

When the red curve is superimposed over the original data (**Figure 1D**), it appears to be an accurate proxy for all of the studies, especially in the lower HbA_1c_ range. The average deviation is very similar to that of the original ‘blue line’ deviation. In the high HbA_1c_ range, the model overestimates against the ‘green line’ deviation but slightly underestimates against the ‘purple line’.

**Figure 1D**

**Appendix II: Effect Ranges**

Deviation of values – effect per 1% HbA_1c_ reduction

| **Complication** | **Min value** | **Max value** | **Average** | **Bottom 50%** | **Top 50%** | **Used** |
| --- | --- | --- | --- | --- | --- | --- |
| **Amputation** | **15.25%** | **60.32%** | **24.94%** | **20.90%** | **49.58%** | **20.63%** |
| T1DM | **15.25%** | **60.32%** | **21.84%** |  |  | 15.25% |
| T2DM | **30.56%** | **43.00%** | **32.23%** |  |  | 30.56% |
|  |  |  |  |  |  |  |
| **Neuropathy** | **14.53%** | **64.09%** | **37.77%** | **30.25%** | **49.78%** | **37.77%** |
| T1DM | 14.53% | 64.09% | 37.77% |  |  |  |
| T2DM |  |  |  |  |  |  |
|  |  |  |  |  |  |  |
| **DKA** | **30.07%** | **40.48%** | **30.07%** | **30.07%** | **40.48%** | **30.07%** |
| T1DM | 30.07% | 40.48% | 30.07% |  |  |  |
| T2DM |  |  |  |  |  |  |
|  |  |  |  |  |  |  |
| **MI** | **9.91%** | **23.08%** | **19.17%** | **12.94%** | **21.72%** | **19.17%** |
| T1DM | 14.10% | 23.08% | 21.95% |  |  | 21.95% |
| T2DM | 9.91% | 15.00% | 13.07% |  |  | 13.07% |
|  |  |  |  |  |  |  |
| **MI - Angina** |  |  | **19.17%** | **12.94%** | **21.72%** | **19.17%** |
| T1DM |  |  | 21.95% |  |  | 21.95% |
| T2DM |  |  | 13.07% |  |  | 13.07% |
|  |  |  |  |  |  |  |
| **MI - CHF** |  |  | **19.17%** | **12.94%** | **21.72%** | **19.17%** |
| T1DM |  |  | 21.95% |  |  | 21.95% |
| T2DM |  |  | 13.07% |  |  | 13.07% |
|  |  |  |  |  |  |  |
| **Nephro: ESRD** | **8.26%** | **23.27%** | **16.72%** | **12.07%** | **21.07%** | **16.72%** |
| T1DM | 11.50% | 60.32% | 21.07% |  |  | 21.07% |
| T2DM | 8.26% | 37.00% | 8.26% |  |  | 8.26% |
|  |  |  |  |  |  |  |
| **Retino: proliferative** | **31.16%** | **59.25%** | **40.86%** | **35.46%** | **46.11%** | **40.86%** |
| T1DM | 31.16% | 59.25% | 40.86% |  |  |  |
| T2DM |  |  |  |  |  |  |
|  |  |  |  |  |  |  |
| **Retino: blindness** | **13.04%** | **60.32%** | **39.14%** | **20.04%** | **47.83%** | **39.14%** |
| T1DM | 13.04% | 60.32% | 40.17% |  |  | 40.17% |
| T2DM | 34.94% | 37.00% | 36.92% |  |  | 36.92% |
|  |  |  |  |  |  |  |
| **Stroke** | **9.09%** | **22.40%** | **14.00%** | **11.11%** | **16.75%** | **14.00%** |
| T1DM | 14.10% | 22.40% | 17.40% |  |  | 17.40% |
| T2DM | 9.09% | 14.53% | 11.89% |  |  | 11.89% |

**Appendix III: Incidence Ranges**

Deviation of values – incidences, original and ‘normalized’ incidences

|  | **Original Incidences** | | | | **Normalized Incidences** | | | |
| --- | --- | --- | --- | --- | --- | --- | --- | --- |
| **Complication** | **Min value** | **Max value** | **Average** | **Used** | **Min value** | **Max value** | **Average** | **Used** |
| **Amputation** | **0.13%** | **0.67%** | **0.356%** | **0.311%** | **0.13%** | **0.42%** | **0.300%** | **0.311%** |
| T1DM | 0.13% | 0.67% | 0.412% |  | 0.13% | 0.42% | 0.297% | 0.320% |
| T2DM | 0.13% | 0.35% | 0.229% |  | 0.13% | 0.42% | 0.261% | 0.310% |
|  |  |  |  |  |  |  |  |  |
| **Neuropathy** | **0.55%** | **6.42%** | **2.681%** | **2.681%** | **0.20%** | **8.59%** | **3.013%** | **3.013%** |
| T1DM | 0.55% | 3.70% | 1.980% |  | 0.20% | 5.03% | 2.140% | 2.140% |
| T2DM | 1.36% | 6.42% | 3.546% |  | 1.36% | 8.59% | 4.087% | 4.087% |
|  |  |  |  |  |  |  |  |  |
| **DKA** | **0.05%** | **5.23%** | **2.239%** | **0.640%** | **0.05%** | **6.06%** | **2.498%** | **0.640%** |
| T1DM | 0.64% | 5.23% | 2.482% |  | 0.64% | 6.06% | 2.770% | 2.770% |
| T2DM | 0.05% | 0.64% | 0.345% |  | 0.05% | 0.64% | 0.345% | 0.640% |
|  |  |  |  |  |  |  |  |  |
| **Hypo (SHE)** | **9.2%** | **390.0%** | **70.7%** | **70.68%** | **8.9%** | **381.8%** | **66.0%** | **66.04%** |
| T1DM | 16.6% | 390.0% | 92.4% |  | 18.26% | 381.8% | 86.0% | 86.0% |
| T2DM | 9.2% | 279.2% | 51.5% |  | 8.9% | 244.6% | 47.7% | 47.7% |
|  |  |  |  |  |  |  |  |  |
| **MI** | **0.50%** | **2.19%** | **1.044%** | **1.044%** | **0.22%** | **2.19%** | **1.114%** | **1.114%** |
| T1DM | 0.52% | 1.20% | 0.828% |  | 0.22% | 1.42% | 0.811% | 0.811% |
| T2DM | 0.50% | 2.19% | 1.116% |  | 0.52% | 2.19% | 1.220% | 1.220% |
|  |  |  |  |  |  |  |  |  |
| **MI - Angina** | **0.88%** | **13.61%** | **3.262%** | **3.262%** | **0.46%** | **13.61%** | **3.261%** | **3.261%** |
| T1DM | 0.88% | 13.61% | 3.241% |  | 0.46% | 13.61% | 3.105% | 3.105% |
| T2DM | 0.98% | 13.61% | 5.356% |  | 1.25% | 13.61% | 5.517% | 5.517% |
|  |  |  |  |  |  |  |  |  |
| **MI - CHF** | **0.34%** | **4.28%** | **1.796%** | **0.840%** | **0.36%** | **4.93%** | **1.908%** | **0.840%** |
| T1DM | 0.34% | 0.84% | 0.591% |  | 0.36% | 0.84% | 0.602% | 0.602% |
| T2DM | 0.84% | 4.28% | 2.088% |  | 0.84% | 4.93% | 2.218% | 2.218% |
|  |  |  |  |  |  |  |  |  |
| **Nephro: ESRD** | **0.04%** | **2.13%** | **0.751%** | **0.751%** | **0.04%** | **1.77%** | **0.705%** | **0.705%** |
| T1DM | 0.04% | 2.13% | 0.677% |  | 0.04% | 1.69% | 0.643% | 0.643% |
| T2DM | 0.50% | 2.04% | 1.271% |  | 0.50% | 1.77% | 1.137% | 1.137% |
|  |  |  |  |  |  |  |  |  |
| **Retino: proliferative** | **0.45%** | **2.76%** | **1.666%** | **1.666%** | **0.49%** | **2.20%** | **1.397%** | **1.397%** |
| T1DM | 0.45% | 2.76% | 1.666% |  | 0.49% | 2.20% | 1.397% |  |
| T2DM |  |  |  |  |  |  |  |  |
|  |  |  |  |  |  |  |  |  |
| **Retino: blindness** | **0.11%** | **0.41%** | **0.225%** | **0.225%** | **0.11%** | **0.41%** | **0.224%** | **0.224%** |
| T1DM | 0.11% | 0.41% | 0.247% |  | 0.11% | 0.41% | 0.246% | 0.246% |
| T2DM | 0.16% | 0.22% | 0.190% |  | 0.16% | 0.22% | 0.190% | 0.190% |
|  |  |  |  |  |  |  |  |  |
| **Stroke** | **0.11%** | **2.29%** | **0.888%** | **0.888%** | **0.11%** | **2.37%** | **0.930%** | **0.930%** |
| T1DM | 0.11% | 1.00% | 0.484% |  | 0.11% | 1.13% | 0.461% | 0.461% |
| T2DM | 0.28% | 2.29% | 1.035% |  | 0.36% | 2.37% | 1.103% | 1.103% |

**Appendix IV: Overview - Risk Deviations**


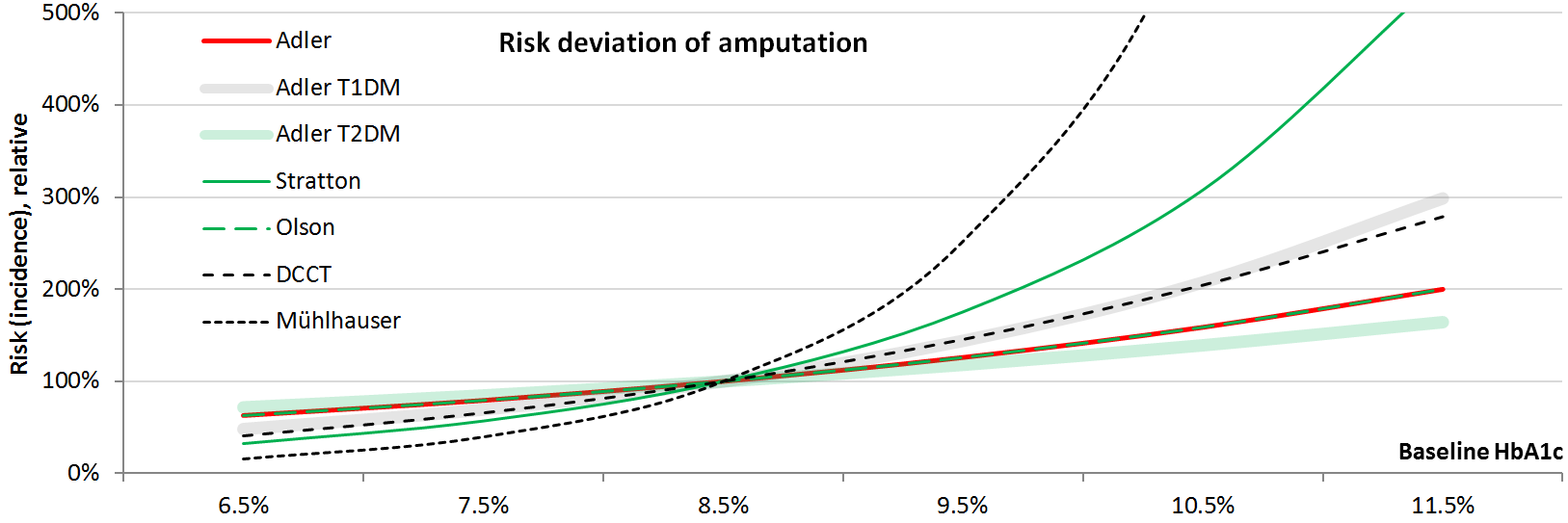


Shows clear underestimation against Stratton (UKPDS) and DCCT with slight underestimation against T1DM studies and slight overestimation against T2DM studies.


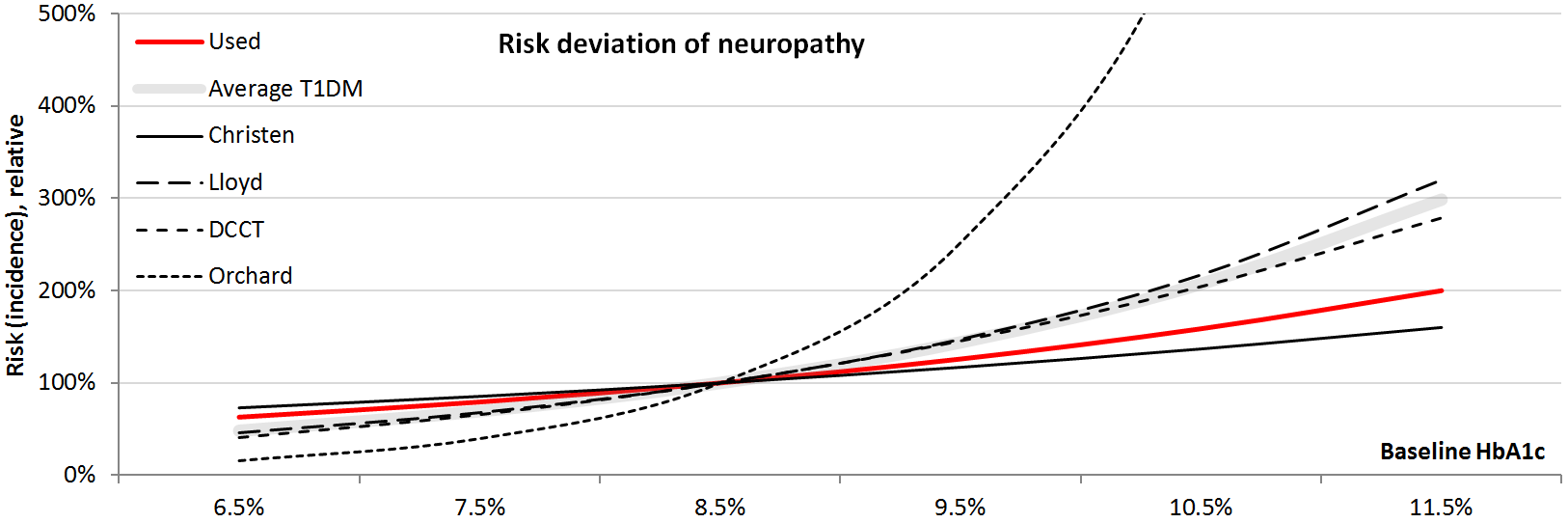


Shows slight underestimation against DCCT and Lloyd with clear underestimation against Orchard


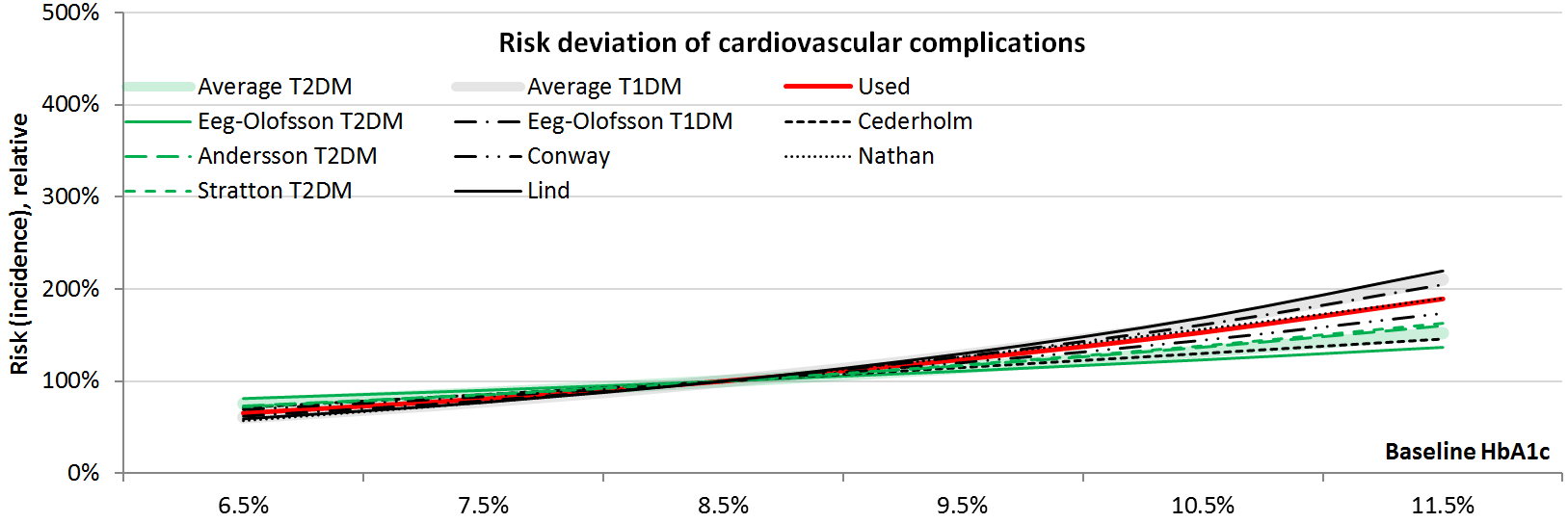


Shows very close fit for all publications with slight underestimation against T1DM studies and slight overestimation against T2DM studies.


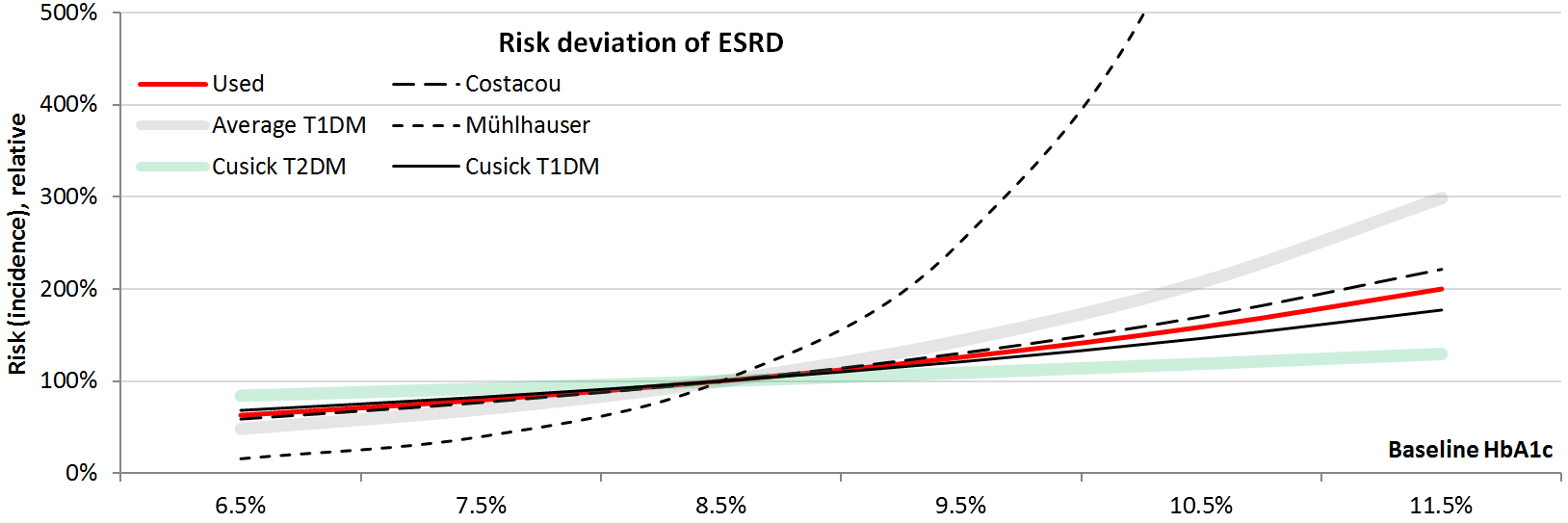


Shows clear underestimation against Mühlhauser but close fit for the other publications. Slight underestimation against T1DM studies with slight overestimation against the type 2 study.


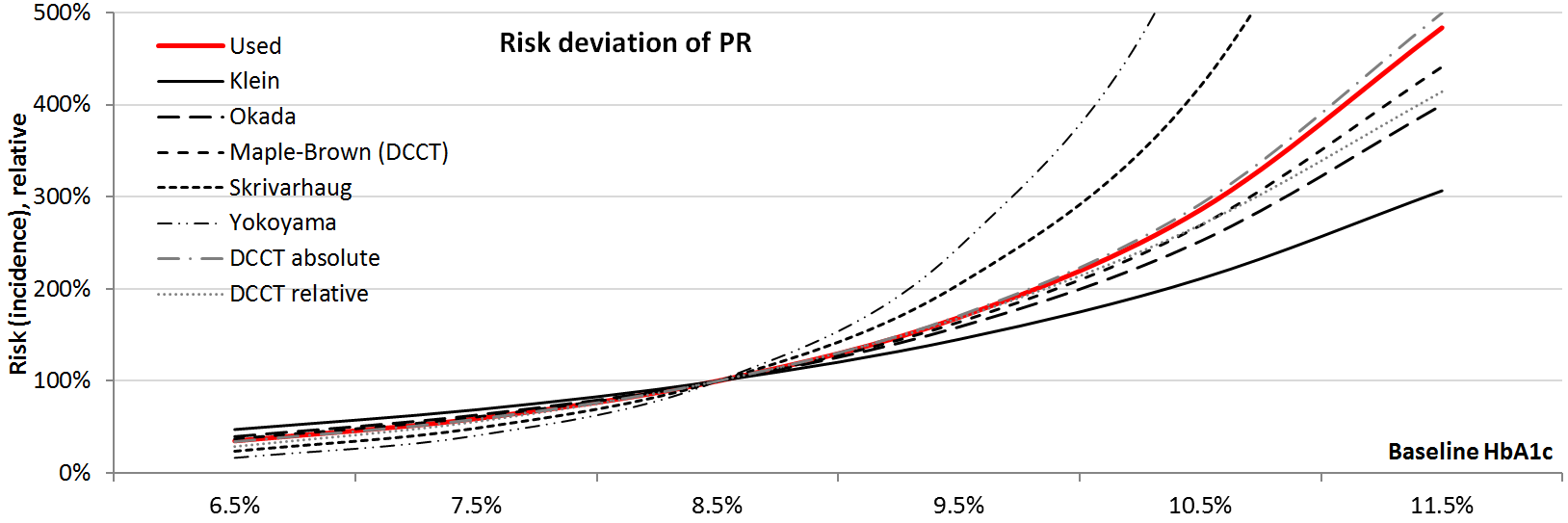


Shows underestimation against Yokohama and Skrivarhaug with overestimation against Klein and close fit to the DCCT publications.


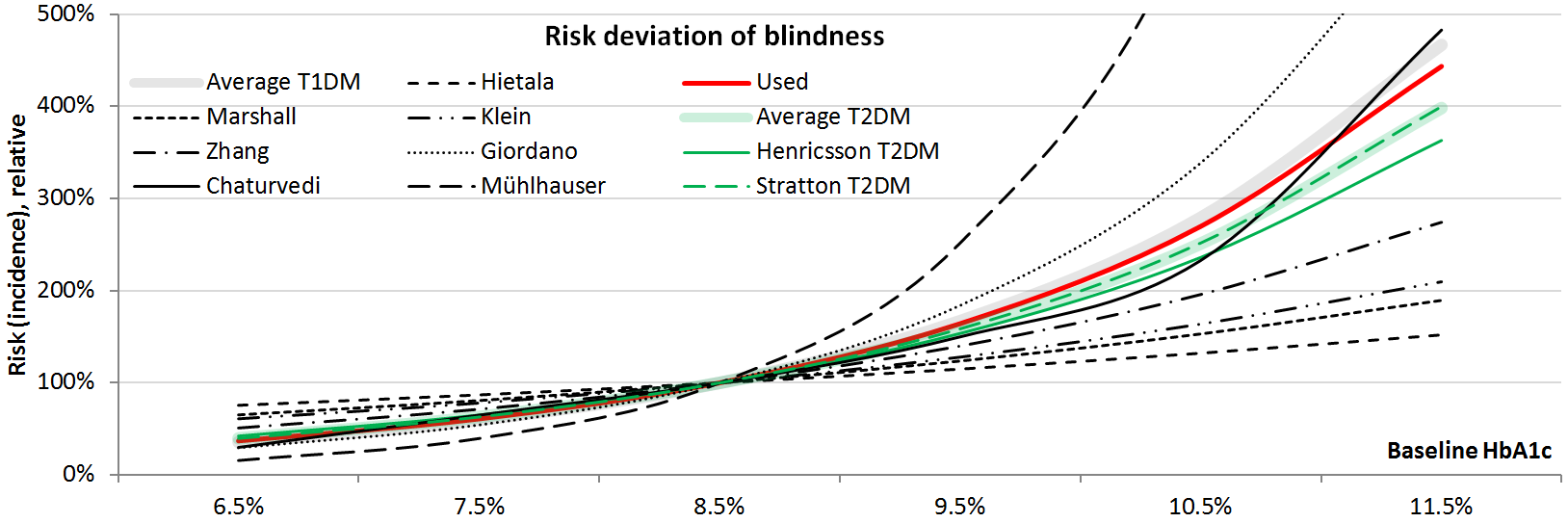


Shows underestimation against Mühlhauser and Giordano with overestimation against Marshall, Klein, Zhang and Hietala. Close fit to the T2DM publications and Chaturvedi. Slight underestimation against T1DM studies and slight overestimation against T2DM studies.


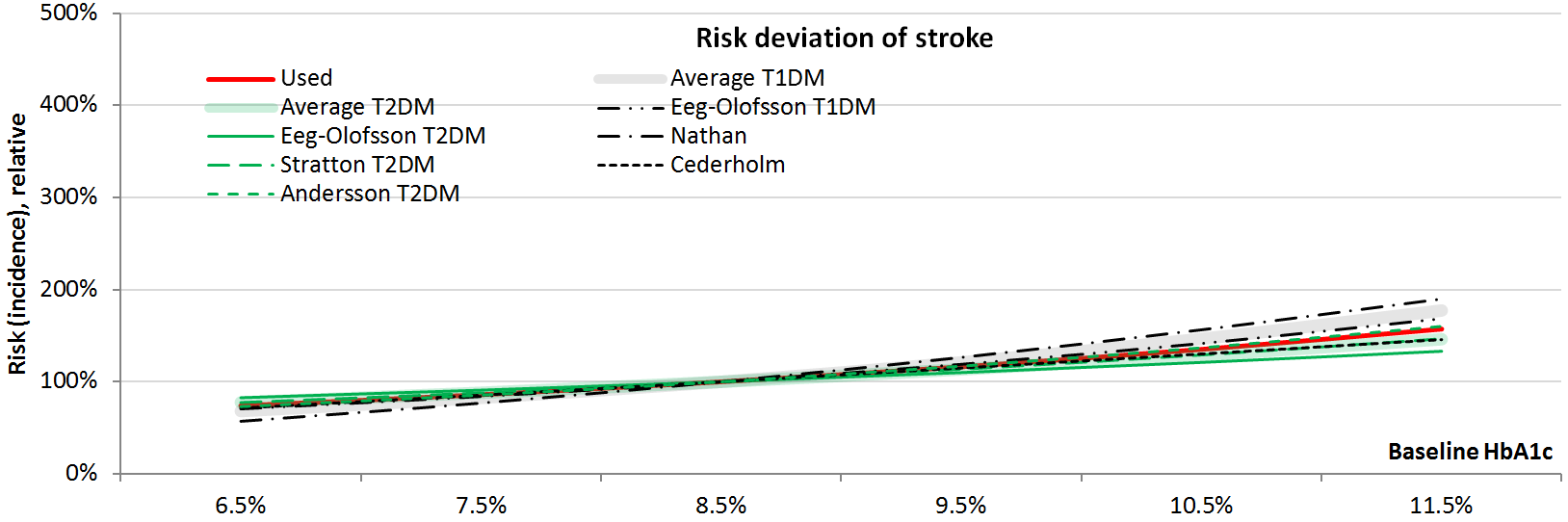


Shows very close fit for all publications with slight underestimation against T1DM studies and slight overestimation against T2DM studies.

**Appendix V: Validation, Further Sensitivity Analyses**

**A. Head to head comparison of the results calculated with the HbA_1c_ Translator with other approaches**

The HbA_1c_ Translator is a model what was generated with the modeling approach. For the following plausibility checks, found averages for incidences and associations were used (see Appendix II and III). How do the results calculated with that model compare to calculations with alternative approaches such as Aagren (1) and Gilmer? (2) To perform the calculations necessary to answer this question, the United States cost dataset (which is most actual), (3) since both Aagren and Gilmer are studies performed in the United States.

Both Aagren and Gilmer show percentage cost differences on the medical or diabetes-related expenditure for populations with different HbA_1c_ values. Those can easily be used for the estimation of cost changes due to changes in HbA_1c_ values (an often used simple alternative to the predictive modeling described here). Percentage and absolute cost changes are a secondary and the main outcome of our modeling approach, so a direct comparison does make sense.

Usage of Aagren or Gilmer for the calculation of predicted cost changes will always lead to an underestimation of the effect of prevented events on the cost (in the retrospective analyses, the therapy cost necessary to reach the HbA_1c_ reduction will reduce the difference in cost). We want to prevent overestimations, underestimations are accepted. So a comparison of our results with such sources is appropriate.

In 2011, Aagren et al. have shown a (relative) cost reduction per 1% (absolute) HbA_1c_ reduction of 5.7% for T1DM and 4.2% for T2DM patients. In 1997, Gilmer et al. have shown a reduction of 6.5% in the overall diabetic population. With the modeling approach, the calculated cost reduction is around 20% (depending on the selected sources). This makes perfect sense: In the modeling approach, only the reduced cost was calculated because of prevented events. The retrospective analyses consider the reduced cost of prevented events, but also the cost of the therapies necessary to reach and maintain the HbA_1c_ reduction. In 1992-1995 (when the Gilmer data was collected), the more effective (but also more expensive) therapies like continuous subcutaneous insulin infusion (CSII), continuous glucose measurement (CGM) or more efficient insulin types were not as common as in 2007, when the Aagren data was collected.

While the relative changes calculated with the HbA_1c_ Translator are much higher than those calculated with Aagren and Gilmer, the absolute differences (the main outcome of our modeling approach) are much closer to the results found with Aagren and Gilmer. That is no surprise since only a subset of all diabetes-related complications are considered. For a population similar to the population observed by Aagren (5% T1DM, average HbA_1c_ value of 7.65%), the expected cost savings of a 1% HbA_1c_ reduction calculated with the HbA_1c_ Translator model are approximately 35% higher than those calculated with Aagren but more than 15% lower as those calculated with Gilmer. For higher baseline HbA_1c_ values, the results of the HbA_1c_ Translator increase more than those calculated with Aagren (which is reflecting the higher relative changes). For populations with only T1DM patients, the results calculated with Aagren approach those calculated with the HbA_1c_ Translator. The relative differences between the HbA_1c_ calculator and Gilmer remain relatively constant (in the range of approximately 15-30%).

|  |  |  | **Generic** | | | **Pure T1DM** | |
| --- | --- | --- | --- | --- | --- | --- | --- |
| **Population:** | **Aagren** | **Gilmer** | **7.5%** | **9%** | **10%** | **Aagren** | **Gilmer** |
| HbA_1c_: | 7.65% | 8.30% | 7.50% | 9.00% | 10.00% | 7.65% | 8.30% |
| T1DM: | 5.06% | 10.00% | 50.00% | 50.00% | 50.00% | 100.00% | 100.00% |
| T2DM: | 94.94% | 90.00% | 50.00% | 50.00% | 50.00% | 0.00% | 0.00% |
| **Results:** |  |  |  |  |  |  |  |
| Aagren: | €313.77 | €328.57 | €360.82 | €385.72 | €403.29 | €418.27 | €430.54 |
| Gilmer: | €505.85 | €642.89 | €477.42 | €796.57 | €900.05 | €505.85 | €642.89 |
| HbA_1c_ Translator (Palmer 2010): | €421.86 | €484.13 | €408.82 | €563.38 | €704.61 | €421.86 | €484.13 |

Table 1: Cost reduction per patient year (PPY) (Euro, 2013 figures) per 1% HbA_1c_ reduction

**B. More than one cost dataset for a country**

More than one source of cost data was found for four countries. When comparing the results of models basing on the different sources, one can see an almost perfect fit for the United Kingdom (4; 5) leading to differences of less than 1%, and two of the three sources with data from the United States (3; 6) lead to differences of only around 1%. For Canada, we see differences of 5-7%.(7; 8) The third dataset from the United States shows relevant structural differences: while the two others have comparable cost for each single complication, this one (9) shows partly extreme differences in both directions. This leads to differences of approx. 1% up to >20%, depending on the baseline HbA_1c_ value. For Sweden, we see an almost constant difference of approx. 30-35%, (10; 11) indicating a structural difference what has to be further investigated. The main difference between the two sources is the diabetes type. In general, sources for T2DM show lower incidence rates but higher cost per complication, leading to almost the same overall cost and cost difference. But the two cost datasets from UK (one for T1DM, one for T2DM patients) show no structural or absolute differences.

All in all, the comparisons show good comparability of the results.

| **Population:** | **Aagren** |  | | **Gilmer** |  | | **Generic** |  | |
| --- | --- | --- | --- | --- | --- | --- | --- | --- | --- |
| HbA_1c_: | 7.65% |  |  | 8.30% |  |  | 9.00% |  |  |
| T1DM: | 5.06% |  |  | 10.00% |  |  | 50.00% |  |  |
| **United States** |  | **Diff. to…** | |  | **Diff. to…** | |  | **Diff. to…** | |
| HbA_1c_ Translator (O'Brien 2003): | €427.02 | **1^st^** |  | €487.93 | **1^st^** |  | €564.62 | **1^st^** |  |
| HbA_1c_ Translator (Pelletier 2008): | €439.72 | 3.0% | **2^nd^** | €529.39 | 8.5% | **2^nd^** | €651.57 | 15.4% | **2^nd^** |
| HbA_1c_ Translator (Palmer 2010): | €421.86 | -1.2% | -4.1% | €484.13 | -0.8% | -8.5% | €563.38 | -0.2% | -13.5% |
| **Sweden** |  |  |  |  |  |  |  |  |  |
| HbA_1c_ Translator (Valentine 2011): | €298.26 | **Diff to 1^st^** | | €346.01 | **Diff to 1^st^** | | €407.99 | **Diff to 1^st^** | |
| HbA_1c_ Translator (Smith-Palmer 2012): | €388.10 | 30.1% |  | €455.80 | 31.7% |  | €545.36 | 33.7% |  |
| **United Kingdom** |  |  |  |  |  |  |  |  |  |
| HbA_1c_ Translator (Roze 2005): | €198.45 | **Diff to 1^st^** | | €230.97 | **Diff to 1^st^** | | €273.36 | **Diff to 1^st^** | |
| HbA_1c_ Translator (Ray 2007): | €200.11 | 0.8% |  | €232.90 | 0.8% |  | €275.64 | 0.8% |  |
| **Canada** |  |  |  |  |  |  |  |  |  |
| HbA_1c_ Translator (O'Brien 2003): | €335.92 | **Diff to 1^st^** | | €381.53 | **Diff to 1^st^** | | €438.21 | **Diff to 1^st^** | |
| HbA_1c_ Translator (Cameron 2009): | €353.84 | 5.3% |  | €404.98 | 6.1% |  | €469.42 | 7.1% |  |

Table 2: Comparison of cost reductions PPY (Euro, 2013 figures) per 1% HbA_1c_ reduction, calculated with different cost datasets from one country

**C. Range of possible results**

The motivation to develop this approach was to minimize the high risk of wrong decision-making when building a simple model. This can lead to extreme or even wrong results. Presented below is the maximal deviation for the default population when using correct data but combining it differently. With incorrect use of the sources or an extreme population, this can become even more problematic (e.g., extreme baseline values, prevalence vs. incidence).

Calculated with the HbA_1c_ Translator, based on German cost data (12)

|  |  | **'Worst case' (always min. figures)** | **Default calculation** | **'Best case' (always max. figures)** |
| --- | --- | --- | --- | --- |
| Events | PPY | 0.021 | 0.122 | 0.414 |
| Prevented events | Absolute [PPY] | 0.002 | 0.018 | 0.082 |
|  | Relative [%] | 9.5% | 14.8% | 19.8% |
| Complication related cost | PPY | € 342 | € 2,685 | € 7,472 |
| Cost reduction | Absolute [PPY] | € 21 | € 276 | € 1,083 |
|  | Relative [%] | 6.1% | 10.3% | 14.5% |

Table 3: Comparison of cost reductions PPY (Euro, 2013 figures) per 0.5% HbA_1c_ reduction, 'Best case' vs. 'Worst case' (calculated with extreme selections of incidences and associations in the HbA_1c_ Translator, based on German cost data (12)

A simple predictive model bears the risk of possible variations of approximately 2,000-5,000%, simply because of the selection of the used evidence, which is not acceptable.

**Appendix VI: Using the modeling approach to get a model - the HbA_1c_ Translator**

When using the modeling approach to create a model, one has to employ the same steps used in traditional modeling approach (e.g., searching for evidence, select the sources fitting to the target population, extract the data, build and validate the model). For most of these steps, the modeling approach described here provides much of the preparation work, which ensures reliability (even under pressure) and leads to a significant simplification of the process. At the end, this should lead to a model of acceptable quality at reasonable effort.

After searching for evidence, the appropriateness of found sources for the current task (e.g., ‘build a model to estimate the budget impact of a study’) for each of the found sources must be checked. Diabetes type, treatment, age, comorbidities and many other factors may be reasons for a disqualification. For example, whereas, the UKPDS(13) with T2DM patients is not eligible for a T1DM population, the DCCT (14) with T1DM patients is not eligible for T2DM patients. The user only can decide this on a case-by-case basis. The proposed modeling approach can support decision-making by providing a comparison of the found data with the range of previously reported values.

Extracting the data from a publication is, in best case, simply reading abstract or results sections. In other cases, the searched value must be extracted from the full text, tables and/or figures. The found figures often must be adapted (e.g., baseline correction, calculate incidence from prevalence, inflate the cost of a publication, conversion of time frames, etc.), which can be very time-consuming and prone to errors. For the evidence underlying the proposed modeling approach (over 100 publications with thousands of figures), data extraction was conducted systematically and with more effort than usually occurs when developing a simple model. Due to our broad approach, there’s a good chance that a publication selected for the current task is already analyzed and the user can use the data extracted by us. This and the comparison with the range of previously reported values may prevent extreme errors like use of prevalence as incidence.

The cost data has the best chance for local evidence. But here, the analysis and adaption of the data is particularly complex. So we did the full analysis and adaptions for as many publications with (almost) complete datasets as possible. All in all, we’ve identified and processed sixteen datasets from eleven countries. So also here, together with our adaption of the cost to other countries according to the WHO figures, we can provide ranges of previously reported cost for each single complication.

When building the model, the approach provides a template to insert the data that is instantly compared with the range of previously reported values. This, plus the provided options for sensitivity analyses, support the validation of the model. The provided example outcomes (figures and graphs) significantly reduce the effort to build the model.

Besides the careful selection of evidence eligible for the model, the analysis of the effect of the modeled therapy on the potential ‘side effect’ of an HbA1c change (e.g., SHE) is crucial for the quality of the model. Most studies report the change in SHE or at least surrogate parameters (like ‘values below 50 mg/dL’) as primary or secondary outcome.

The HbA_1c_ Translator interface, based upon the modeling approach described, is designed for ease of use and flexibility and is intended for use in insulin-treated diabetes patients.


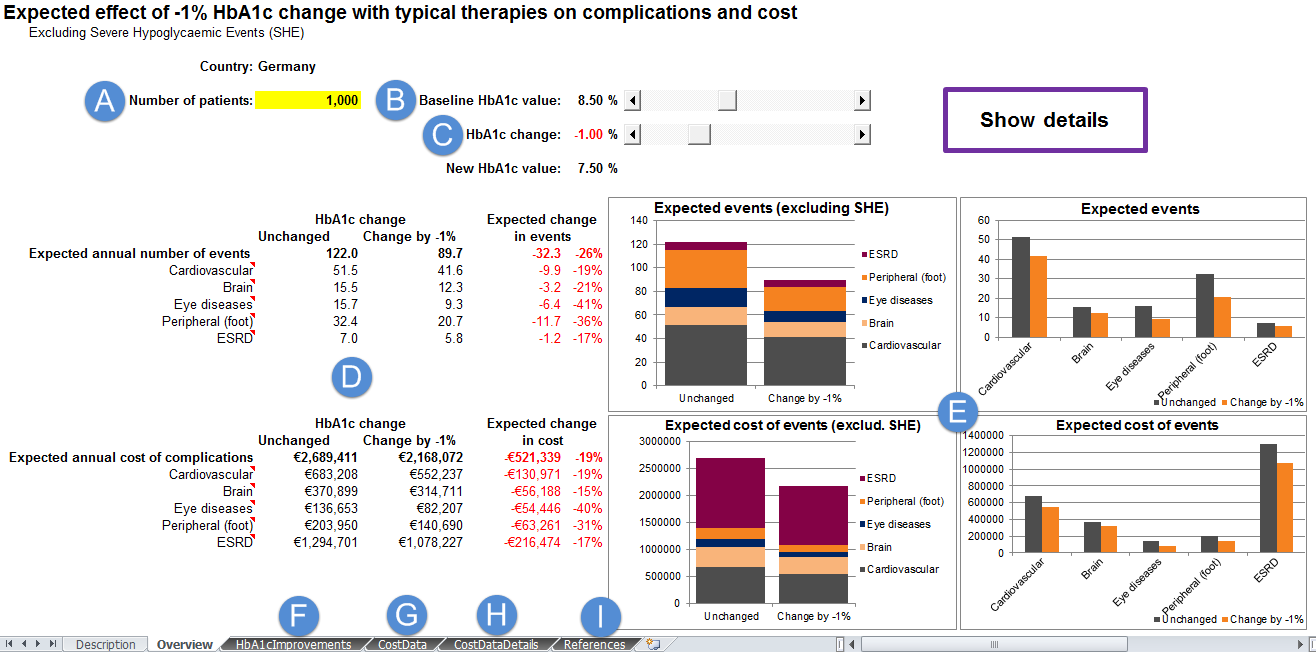


When accessing the model, the default values are presented first. The user then modifies the number of patients (**A**), baseline HbA_1c_ for the population studied (**B**) and projected change in HbA_1c_ (**C**). Once these parameters have been modified, the model calculates the potential change (number/percentage) in events and related costs (**D**). The expected events and costs are graphed per complication and proportionally (**E**). Tabs at the bottom of the interface page allow users to access and modify all data utilized in the calculations, including incidences, deviation and cost of incidences (**F**), costs by country (**G**) and cost data inputs (**H**). Users can also access web-linked references for all studies utilized in the model, which further enhances model transparency (**I**).

*References*

1. Aagren M, Luo W: Association between glycemic control and short-term healthcare costs among commercially insured diabetes patients in the United States. J Med Econ 2011;14:108-114

2. Gilmer TP, O'Connor PJ, Manning WG, Rush WA: The cost to health plans of poor glycemic control. Diabetes Care 1997;20:1847-1853

3. Palmer JL, Knudsen MS, Aagren M, Thomsen TL: Cost-effectiveness of switching to biphasic insulin aspart from human premix insulin in a US setting. J Med Econ 2010;13:212-220

4. Roze S, Valentine WJ, Zakrzewska KE, Palmer AJ: Health-economic comparison of continuous subcutaneous insulin infusion with multiple daily injection for the treatment of Type 1 diabetes in the UK. Diabet Med 2005;22:1239-1245

5. Ray JA, Boye KS, Yurgin N, Valentine WJ, Roze S, McKendrick J, Tucker DM, Foos V, Palmer AJ: Exenatide versus insulin glargine in patients with type 2 diabetes in the UK: a model of long-term clinical and cost outcomes. Curr Med Res Opin 2007;23:609-622

6. O'Brien JA, Patrick AR, Caro J: Estimates of direct medical costs for microvascular and macrovascular complications resulting from type 2 diabetes mellitus in the United States in 2000. Clin Ther 2003;25:1017-1038

7. O'Brien JA, Patrick AR, Caro JJ: Cost of managing complications resulting from type 2 diabetes mellitus in Canada. BMC Health Serv Res 2003;3:7

8. Cameron CG, Bennett HA: Cost-effectiveness of insulin analogues for diabetes mellitus. CMAJ 2009;180:400-407

9. Pelletier EM, Smith PJ, Boye KS, Misurski DA, Tunis SL, Minshall ME: Direct medical costs for type 2 diabetes mellitus complications in the US commercial payer setting: a resource for economic research. Appl Health Econ Health Policy 2008;6:103-112

10. Valentine WJ, Aagren M, Haglund M, Ericsson A, Gschwend MH: Evaluation of the long-term cost-effectiveness of insulin detemir compared with neutral protamine hagedorn insulin in patients with type 1 diabetes using a basal-bolus regimen in Sweden. Scandinavian journal of public health 2011;39:79-87

11. Smith-Palmer J, Fajardo-Montanana C, Pollock RF, Ericsson A, Valentine WJ: Long-term cost-effectiveness of insulin detemir versus NPH insulin in type 2 diabetes in Sweden. J Med Econ 2012;15:977-986

12. Valentine WJ, Goodall G, Aagren M, Nielsen S, Palmer AJ, Erny-Albrecht K: Evaluating the cost-effectiveness of therapy conversion to insulin detemir in patients with type 2 diabetes in Germany: a modelling study of long-term clinical and cost outcomes. Advances in therapy 2008;25:567-584

13. Stratton IM, Adler AI, Neil HA, et al. Association of glycaemia with macrovascular and microvascular complications of type 2 diabetes (UKPDS 35): prospective observational study. BMJ. 2000;321(7258):405-412.

14. Nathan DM, Cleary PA, Backlund JY, et al. Intensive diabetes treatment and cardiovascular disease in patients with type 1 diabetes. N Engl J Med. 2005;353(25):2643-2653.
